# Long-Term Survival of a Patient With Locally Advanced Esophageal Cancer Treated With Chemoradiotherapy Following Esophageal Bypass Surgery: A Case Report

**DOI:** 10.7759/cureus.92084

**Published:** 2025-09-11

**Authors:** Masataka Ando, Takuya Saito, Kentaro Shinohara, Yasuyuki Fukami, Tsuyoshi Sano

**Affiliations:** 1 Division of Gastroenterological Surgery, Department of Surgery, Aichi Medical University, Nagakute, JPN

**Keywords:** bypass surgery, chemoradiotherapy, esophageal cancer, long-term survival, unresectable

## Abstract

Esophageal bypass surgery is a palliative option for patients with unresectable esophageal cancer who present with severe obstruction or esophago-respiratory fistula. Although it is highly invasive and associated with frequent postoperative complications, in selected cases, it may support completion of curative-intent therapies. We present a rare case of long-term survival after esophageal bypass performed prior to definitive chemoradiotherapy (CRT).

A 55-year-old woman presented with progressive dysphagia. Endoscopy revealed a circumferential type 2 mass in the mid-esophagus, which could not be traversed. Biopsy confirmed squamous cell carcinoma. Computed tomography (CT) demonstrated a 4 cm mass just below the tracheal bifurcation with suspected bilateral bronchial invasion and multiple mediastinal nodal metastases, staged as cT4bN2M0 (stage IVA). Gastrostomy was initially performed due to poor oral intake, followed by two cycles of docetaxel, cisplatin, and 5-fluorouracil (DCF) as neoadjuvant chemotherapy. Although tumor shrinkage was achieved, residual bronchial invasion rendered the lesion unresectable. To secure oral intake and maintain performance status, laparoscope-assisted esophageal bypass with a cervical gastric tube and Roux-en-Y reconstruction (modified Kirschner procedure) was performed. The postoperative course was uneventful. One month later, the patient underwent definitive CRT (50 Gy with concurrent cisplatin and 5-fluorouracil), followed by four additional cycles of chemotherapy. A complete clinical response was confirmed, and the patient remains disease-free at five years after treatment. Esophageal bypass surgery, when carefully indicated in patients with good performance status, can enable continuation of CRT by stabilizing oral intake and preserving quality of life. Although bypass does not directly improve oncologic outcomes, in selected cases, it may indirectly contribute to long-term survival by supporting completion of curative treatment.

## Introduction

Locally advanced esophageal cancer with direct invasion into adjacent structures (cT4) is frequently unresectable, with an incidence of approximately 12% among all patients with esophageal cancer in Japan [1]. Obstruction and esophago-respiratory fistulae are common complications, leading to malnutrition, impaired quality of life, and difficulty completing chemoradiotherapy (CRT). Esophageal stenting has become the preferred palliative measure due to its minimally invasive nature, yet bypass surgery remains an option in selected patients, particularly where stenting is technically difficult or unsafe. We report a case of long-term survival in a patient who underwent esophageal bypass prior to CRT.

## Case presentation

**Figure 1:**
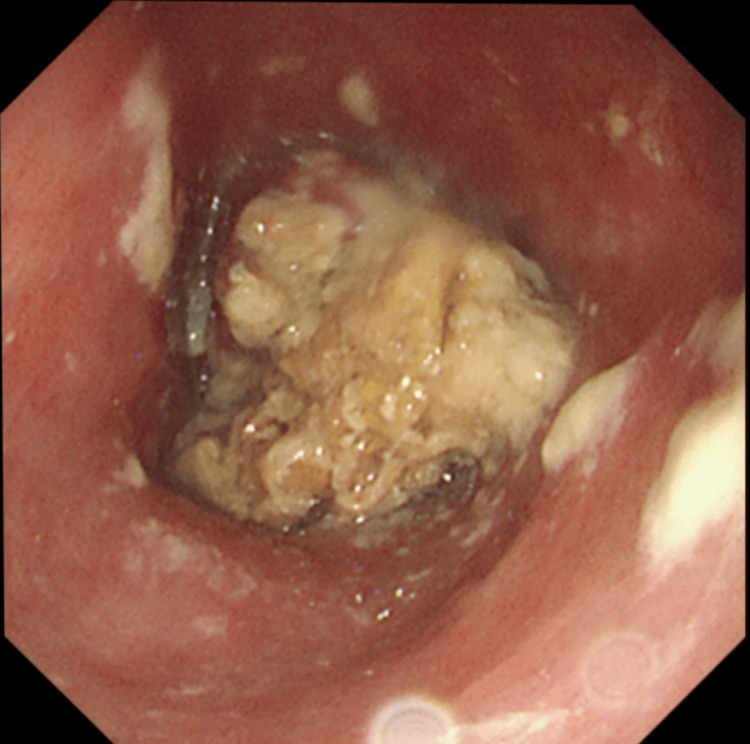
Endoscopic image Circumferential mid-esophageal tumor obstructing passage.

**Figure 2:**
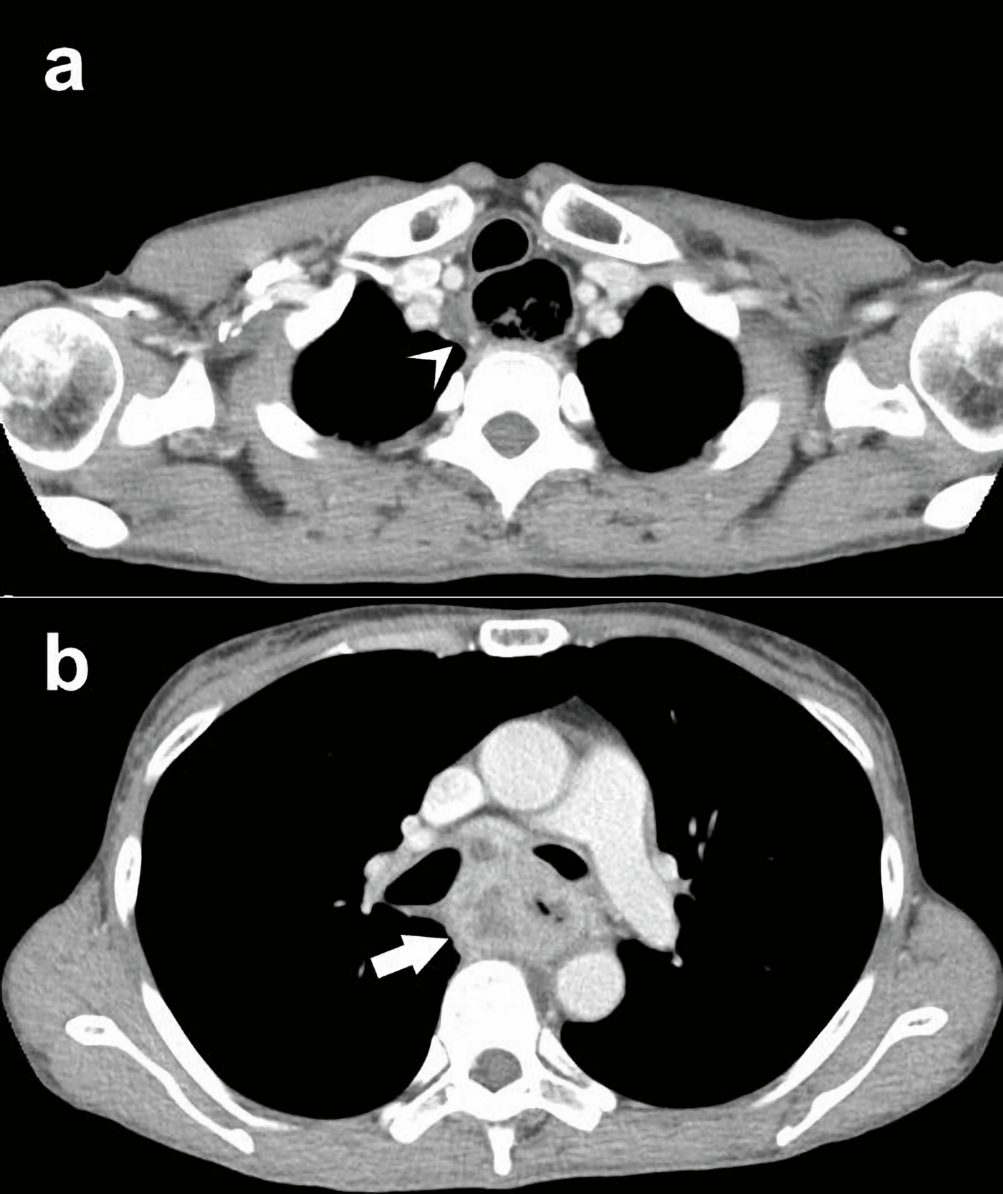
CT findings before treatment (a) Recurrent nerve node metastasis (arrowhead) and esophageal dilatation. (b) 4 cm esophageal mass with suspected bronchial invasion (arrow). Staging was determined according to the TNM Classification of Malignant Tumours, 8th Edition [2]. CT: computed tomography

A 55-year-old woman presented with dysphagia and poor oral intake. Endoscopy revealed a circumferential obstructing tumor in the middle thoracic esophagus (Figure 1). Biopsy confirmed squamous cell carcinoma. Imaging demonstrated a 4 cm tumor with suspected bilateral bronchial invasion and multiple mediastinal nodal metastases without distant disease (Figure 2), staged as cT4bN2M0 (stage IVA) [2].

**Figure 3:**
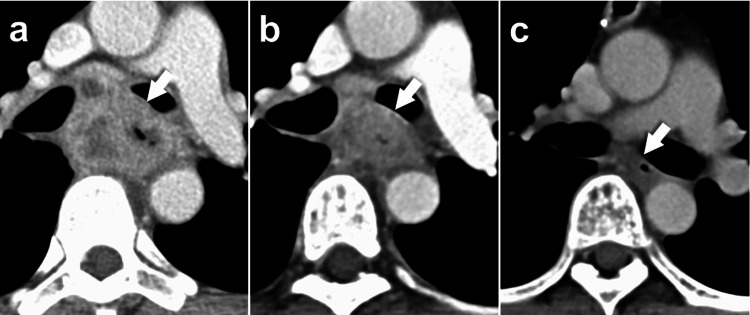
Changes in CT findings associated with treatment (a) Pre-treatment. (b) Post-DCF showing partial shrinkage but residual invasion. (c) Post-CRT and additional chemotherapy showing complete response. Arrows indicate tumor location. Chemotherapy regimen based on Hara et al. [3], and CRT protocol based on Cooper et al. [5]. CT: computed tomography, DCF: docetaxel, cisplatin, and 5-fluorouracil, CRT: chemoradiotherapy

**Figure 4:**
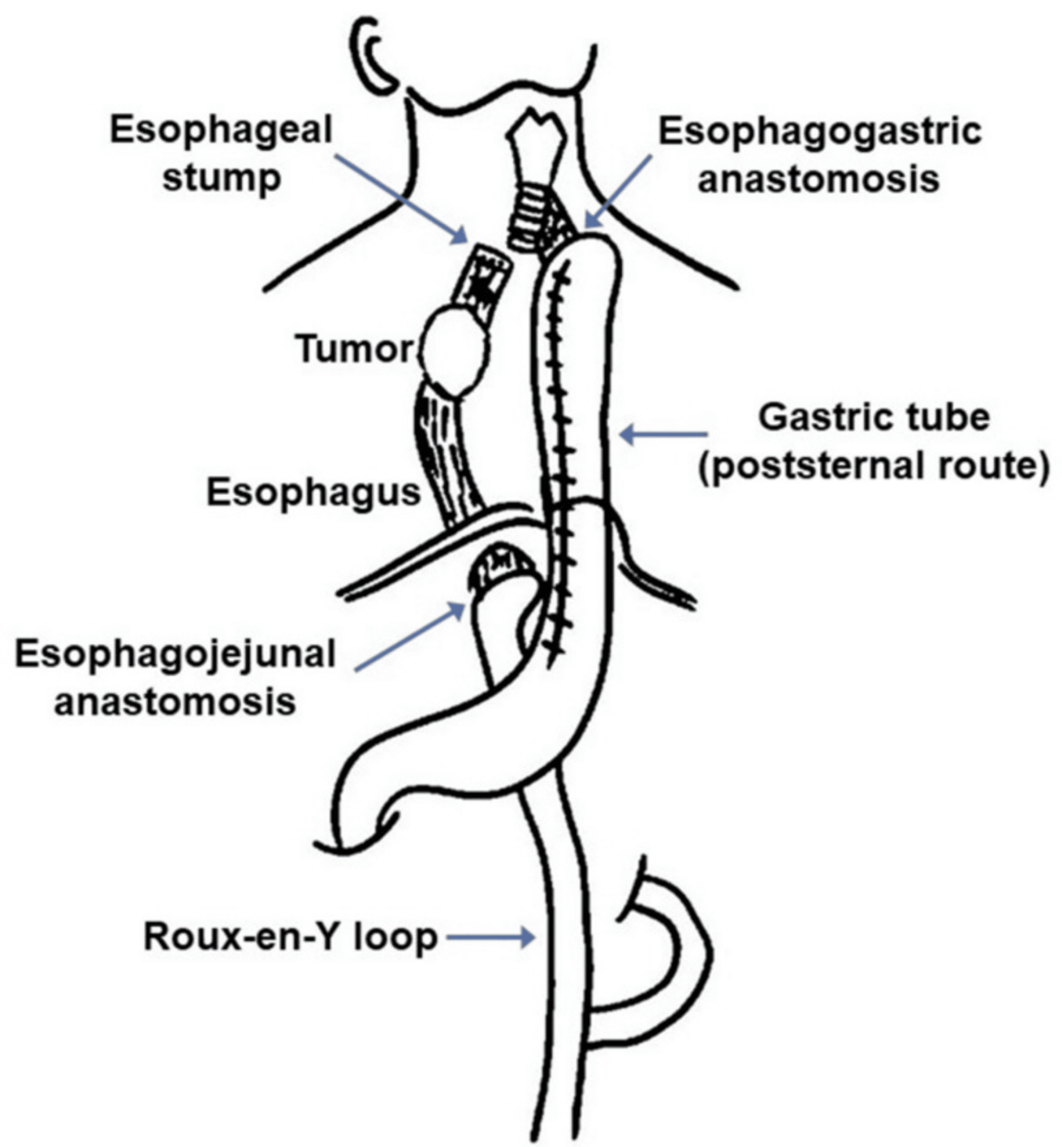
Schema of modified Kirschner operation Gastric tube anastomosis and Roux-en-Y diversion. This figure was created by the authors, based on the method described by Mannell [4].

A gastrostomy was placed for nutritional support, and two cycles of docetaxel, cisplatin, and 5-fluorouracil (DCF) were administered as neoadjuvant chemotherapy [3]. Tumor shrinkage was achieved, but bronchial invasion persisted (Figure 3b), precluding radical resection. To secure oral intake and preserve performance status for further therapy, laparoscope-assisted esophageal bypass was performed. A gastric tube was brought retrosternally to the neck and anastomosed to the proximal esophagus, while the distal esophagus was diverted into a Roux-en-Y jejunal loop (modified Kirschner operation [4]) (Figure 4). The operative time was five hours and 24 minutes with blood loss of 245 mL. The postoperative course was uneventful, and the patient resumed oral intake by day 10.

One month postoperatively, definitive CRT was initiated, consisting of 50 Gy in 25 fractions with concurrent cisplatin and 5-fluorouracil [5]. Four additional cycles of chemotherapy were administered. Six months later, CT demonstrated the disappearance of the primary tumor and nodal disease (Figure 3c). The patient has remained alive without recurrence for five years since initiation of treatment.

## Discussion

Esophageal bypass surgery is a highly invasive palliative procedure with reported complication rates of 32%-53% [6]. Although its use has declined with the availability of stents [7], bypass may be indicated in situations where stenting is not feasible, such as complete obstruction, high migration risk, or large fistulae. Importantly, radiation therapy after stenting is associated with severe complications, including bleeding and pneumonia, making bypass a safer alternative when CRT is planned [8].

Definitive CRT is the first-line treatment for unresectable esophageal cancer, achieving complete response rates of 24%-32% and five-year survival rates of 7%-20% [9]. Fistula formation remains a major complication, occurring in 22%-31% of cases and significantly impairing prognosis [10]. Previous reports have suggested that esophageal bypass prior to CRT may be performed safely in carefully selected patients and can help maintain nutrition and quality of life during therapy [11,12].

At our institution, bypass is considered in patients with complete obstruction where stenting is technically challenging or unsafe, provided performance status is favorable. In the present case, bypass facilitated uninterrupted CRT and subsequent chemotherapy, resulting in a rare long-term disease-free survival.

This case illustrates that while bypass itself does not exert oncologic effects, it may indirectly contribute to improved outcomes by enabling treatment completion. The rarity of such long-term survival highlights the potential role of bypass in selected patients.

## Conclusions

In patients with locally advanced unresectable esophageal cancer and poor oral intake, esophageal bypass may be an option to secure nutrition and support treatment completion when stenting is unsuitable. Although invasive, when performed in carefully selected patients, bypass can maintain quality of life and indirectly contribute to long-term survival. This case illustrates that bypass surgery, while not oncologically curative in itself, may play a critical supportive role in enabling patients to complete definitive chemoradiotherapy and achieve favorable long-term outcomes. Further studies are warranted to clarify its indications and long-term benefits within multidisciplinary treatment strategies.
